# Massage in England in 1913

**Published:** 1913-12-06

**Authors:** 


### MASSAGE IN ENGLAND IN 1913.

### The New Surveillance of Places of Training.

At the present moment attention is being drawn to massage by the proposal before the London County Council for the inspection of nursing and lying-in homes and establishments for massage. This new surveillance will be attended by a good deal of difficulty, and is sure to encounter opposition from some places; but the result ought to be the removal of much that is undesirable, as well as much that is really evil; all those who are honestly anxious for the welfare of the profession of massage will welcome any scheme that will advance and safeguard it.

The Incorporated Society of Trained Masseuses, founded in 1894, was started with the object of raising the standard of massage, protecting a valuable profession, and safeguarding its members from misrepresentation. Further, to provide for and arrange the examination of candidates; to grant certificates to pupils who come from approved schools (*i.e.* from schools whose curriculum is lengthy and thorough enough to be accepted by the Society), and who pass its examinations; to supply lectures and a reference library; and to afford information, advice, and help to members.

The medical and legal professions gave much help to the Society, the management of which is in the hands of a council and an executive committee. The rules formed twenty years ago have proved their value and are as follows:—

1. Not to undertake any cases of massage except under the direction of a registered medical practitioner, and, in regard to massage for men, to act in accordance with the by-laws of the Society.
2. Not to advertise in any way whatever, except in recognised medical papers.
3. Not to sell goods to patients in a professional capacity.

All candidates for examination must sign these rules and undertake to return their certificate if the council considers they are guilty of conduct, professional or otherwise, injurious to the objects of the Society. On these lines the Incorporated Society has worked, and, having begun on a very small scale, has now 2,497 certificate holders on its books; 300 candidates came up for the half-yearly examination held last July, so that it can speak with the authority of long and wide experience.

The Council have devoted much attention to the training of candidates, and consider that after the due regard of physique, intelligence, and personality of the would-be masseuse (which must all be suitable to the work), the actual training should occupy *not less* than six months for massage only, and should include such a course as the following, or an equivalent amount of instruction if it is differently arranged:—

### Lectures on Anatomy.

For instance, six on the skeleton, six on joints, twelve on muscles, eight on nerves, six or eight on blood-vessels and lymphatics.

### Lectures on Physiology.

Say, twelve on an elementary knowledge of the subject.

### Lectures on the Theory of Massage.

At least thirty on the movements used in massage and their uses.

### Lectures on Remedial Exercises.

About twelve on active and passive movements and the ordinary exercises necessary for cases.

Besides these sets of lectures, there must be daily practical work for some hours on living models, and in hospital or some other clinic when the students are more advanced and fitted to treat actual patients under supervision. Bandaging and the use of splints must also be learnt, and papers set and corrected by the teachers on anatomy, physiology, and the theory of massage. The whole of the student's time for six or eight months must be devoted to work, and she should then be fit to pass the examination of the Incorporated Society.

It is, further, most desirable for the student to take a course on the use of batteries, and those who are not nurses should, at all events, hold the first aid and home nursing certificates of the St. John Ambulance Association, which will together occupy another three or four months. To be fully equipped there remains training in medical gymnastics, for which another six months' study will be necessary, and for which the Society also holds an examination, but this examination is only open to candidates who hold its certificate for massage, or some other certificate which is approved by the council.

This amount of teaching, therefore, represents from fifteen to eighteen months of hard work, and constitutes a thorough training. Its expense is comparatively small; £15 for the first six months and £25 for the second will about cover it, exclusive of the cost of living for the time, of some books, examination fees, railway fares, and £5 or £6 for extra subjects, such as electricity. This compares favourably with the cost of most professions.

The work when obtained is well paid, but anyone entering upon it should have some reasonable hope of obtaining it, either from the medical profession or from a hospital or school where she is likely to get an appointment. The Colonies offer a field for work, but full inquiry must be made before going out as the restrictions and requirements vary greatly. The large incomes which have been mentioned in some advertisements are quite beyond the bounds of probability; there are many women who are engaged in this useful and deeply interesting work who are earning from £100 to £300 a year, but it must be remembered that no one reaches the top of the ladder at once, and that the first step is the difficulty.

The word Swedish has become so associated with massage that it is as well to give some account of it as taught in Sweden. There are two great schools recognised by Government: the Central Institute and Dr. Arvedson's School, both in Stockholm. The first only admits Englishwomen who hold a medical degree; this, of course, excludes the majority of candidates from this Institute. At Dr. Arvedson's School pupils are received on three different scales, the first being for qualified doctors and certificated gymnasts who may enter for a short course. This is not regarded as a training, but merely as extra teaching. Beginners are not taken for less time than a year, and if they can work at a high pressure at the end of that time they can obtain a certificate, but this year, owing to the arrangements for holidays, the course consists of eight months' work.

The time required for full training both in medical and school work is two years—*i.e.*, a second year's, or rather eight months' course, which is a repetition of the first eight months' teaching, no fresh subjects being added to the time-table. At the end of the second year students go up for examination and the certificate gained is *official* and gives the title in Sweden of "gymnastik director." To gain this certificate students must be under thirty years of age. The cost is about £45 for the first eight months' instruction and £36 for the second term, exclusive of journeys, books, and living. Students only going for massage and remedial exercises, not scholastic work, may go for a special course from September to May at a charge of about £42. Board and lodging may be obtained for £6 or £7 a month. The diet differs, of course, from English food, and some people might not like it, nor the unaccustomed hours for meals. And there is the real difficulty of learning a subject in an unknown language, so that to benefit by the instruction it is most advisable to acquire some knowledge of Swedish, either by learning in England or by going to Sweden for three months previous to beginning the course.

Much more time is devoted to exercises than to actual massage, which fact is borne out by the experience of the examiners of the Incorporated Society, who find that candidates from English training schools show more finished and careful massage. The Swedish training does not give opportunity for any nursing experience, no patients being treated in bed, and no temperatures being taken or bandaging instruction given. This information is derived from Sweden, and the training of the two countries and their respective merits can be weighed by intending candidates.

There remains one point. Why should not *English* massage be given the first place in *England*? Excellent training, ample hospital experience, and moderate fees are all available, some London hospitals offering an admirable training with every advantage of remedial and clinical work and provision for board and lodging for about £50—£60 for a full year's work. Surely patriotism might be considered, and by the medical profession as well as by the laity.

The members of the Incorporated Society have voluntarily tied their hands in refusing to work without doctors' orders, and it would be an immense help and encouragement if the medical profession would act on the same lines, decline to employ those who undertake cases without any permission from doctors, a practice that is more dangerous than is commonly known.

To return to inspection. Much light may be thrown on the methods followed in many instances when this is inaugurated, and we can only hope that it may be carried out with a thoroughness and discretion which will remove slackness and scandal, raise the standard of massage, and protect the public. The aims of the Incorporated Society would be strongly supported by such action.

